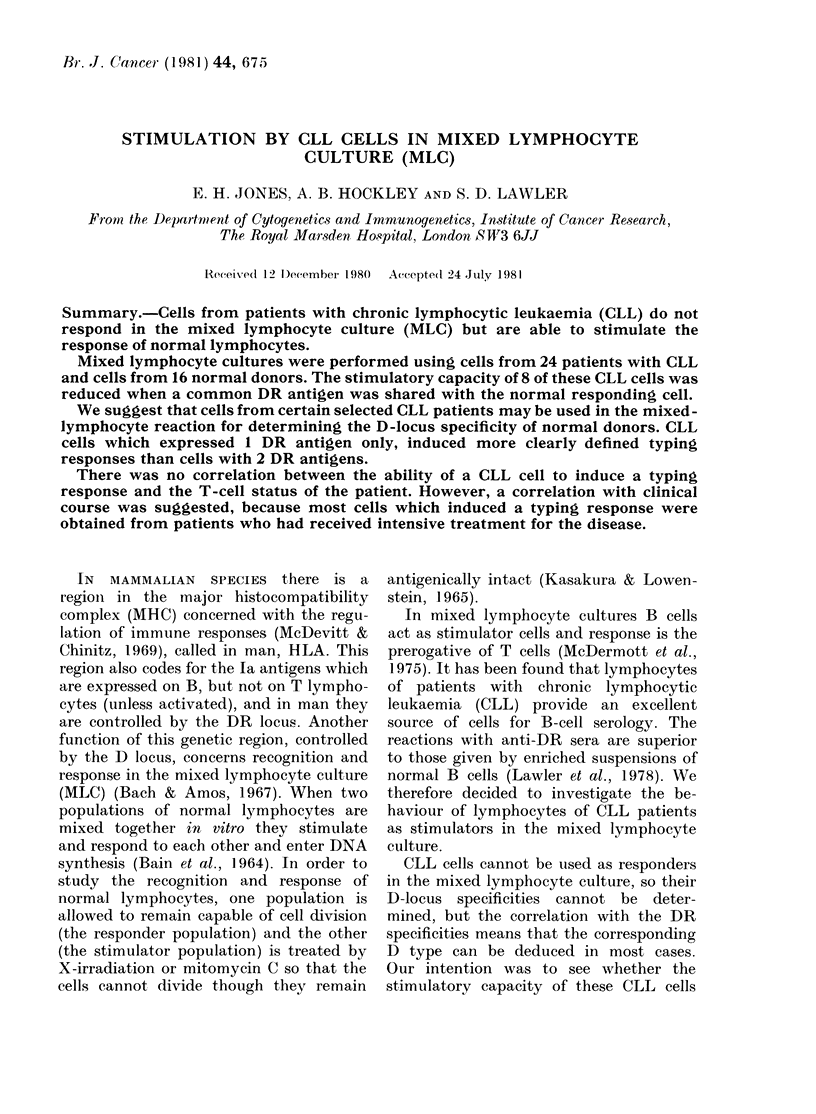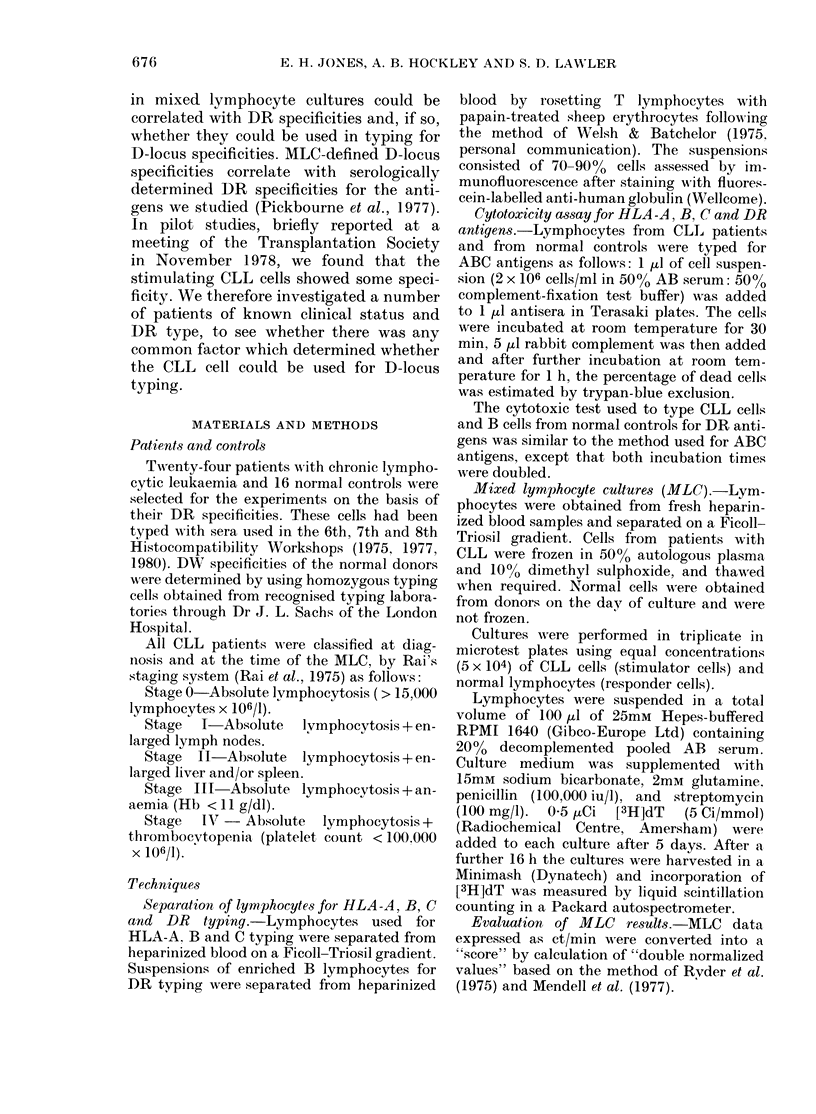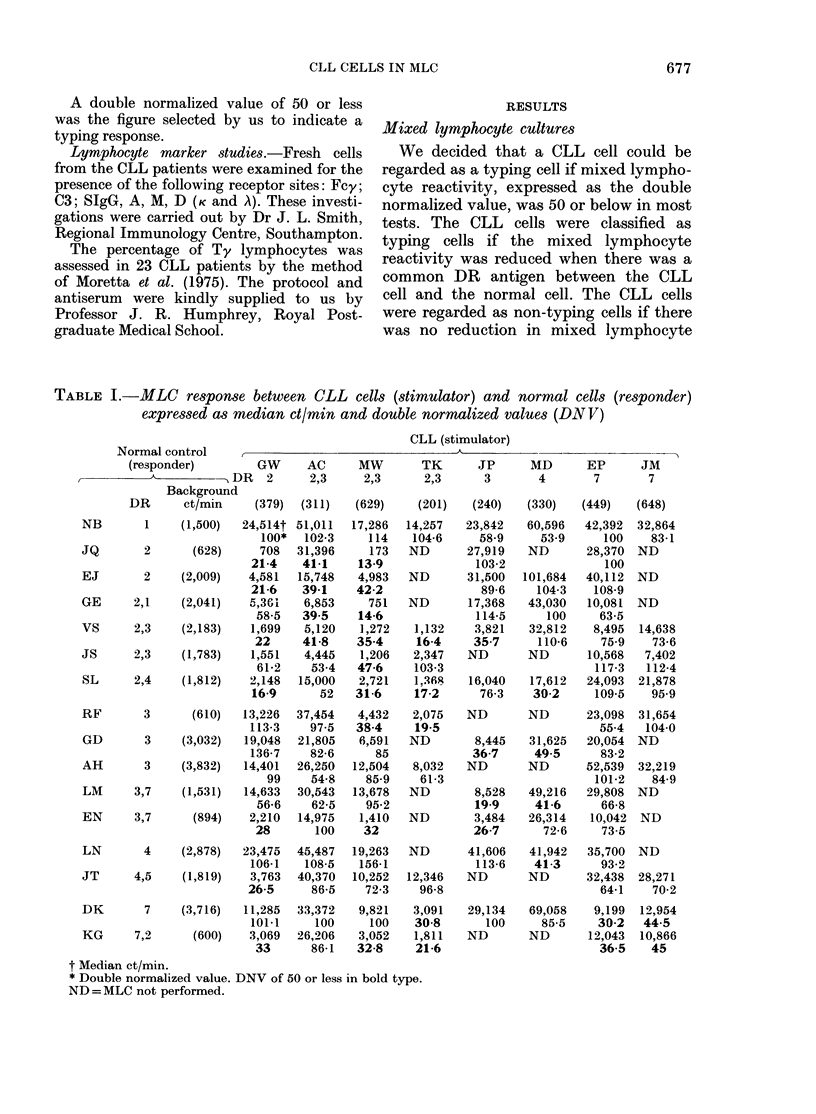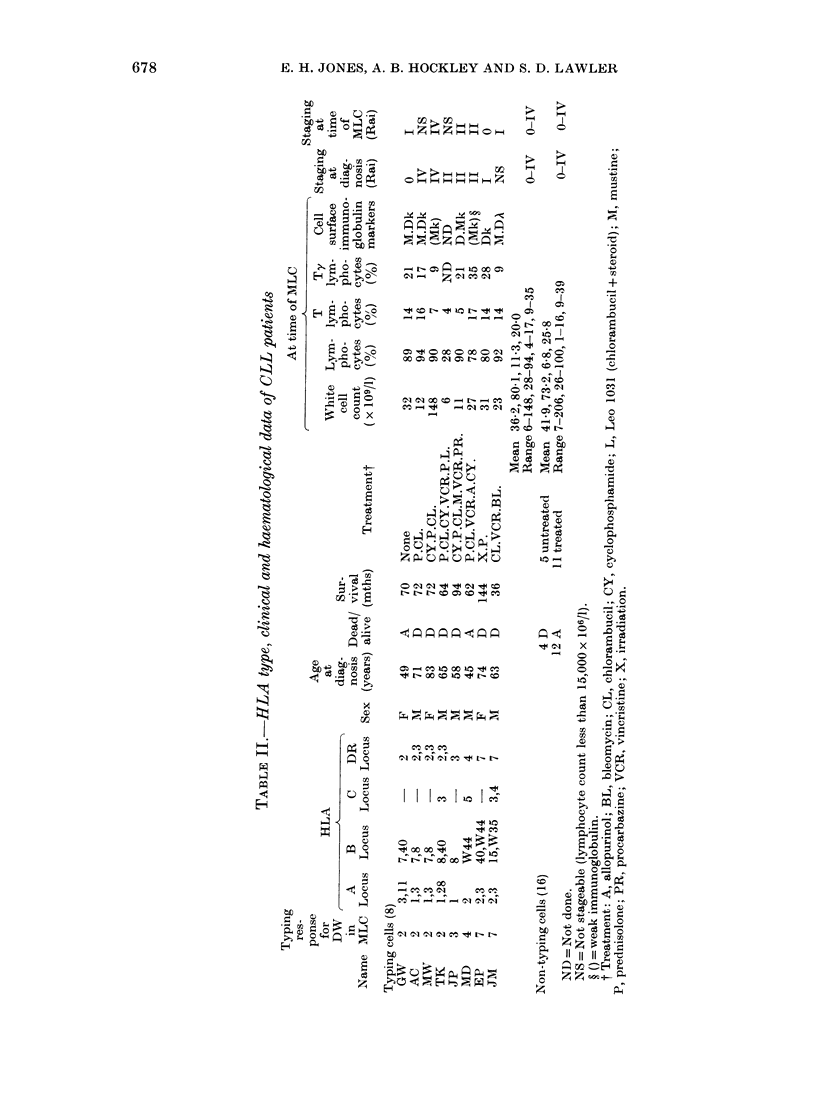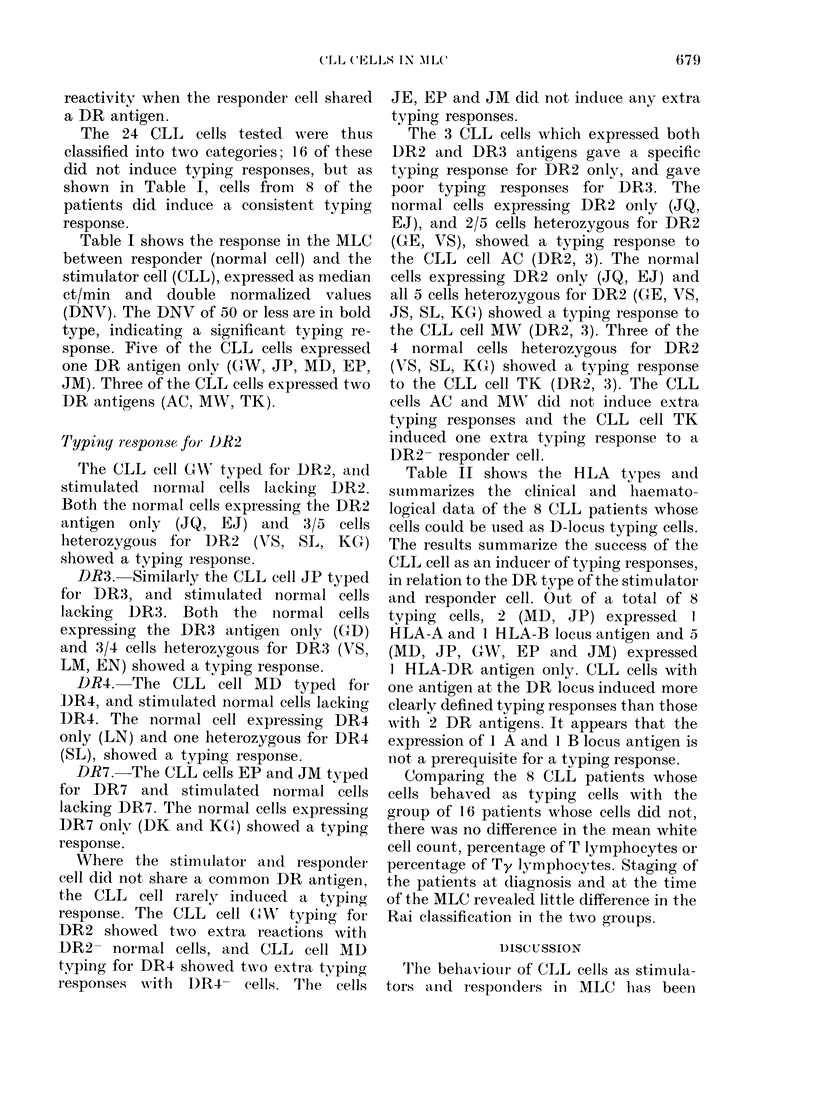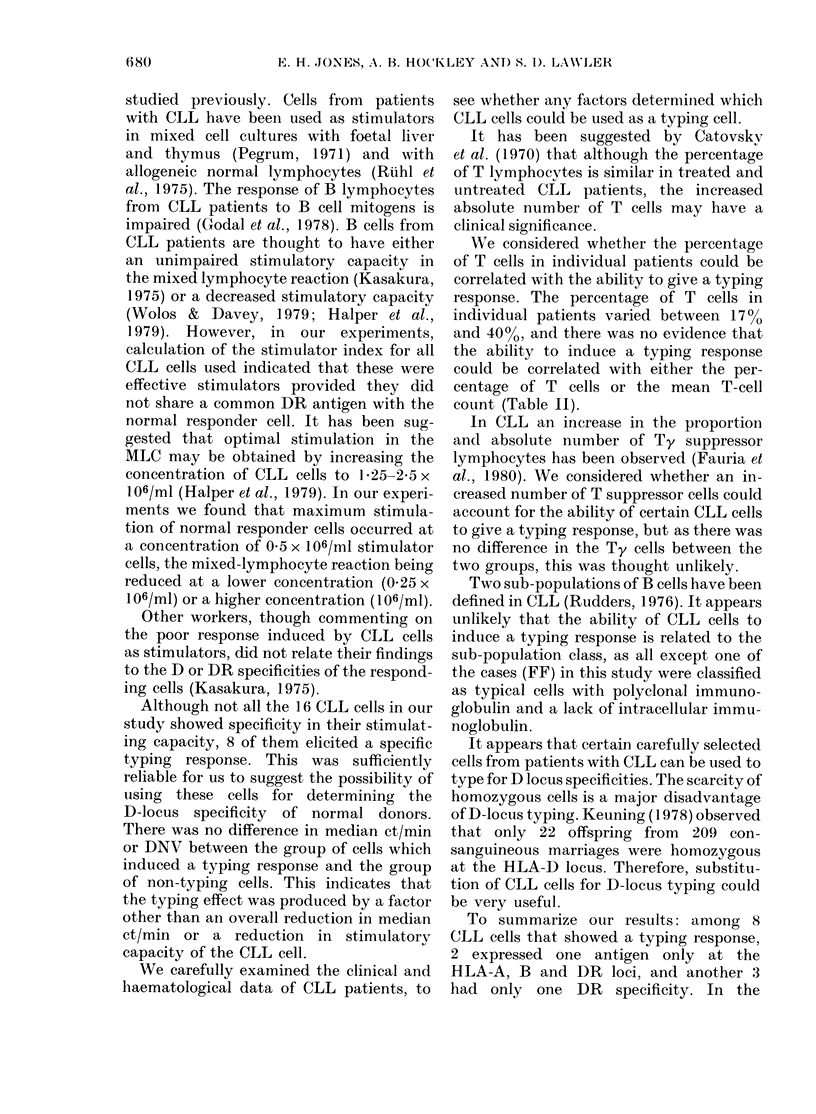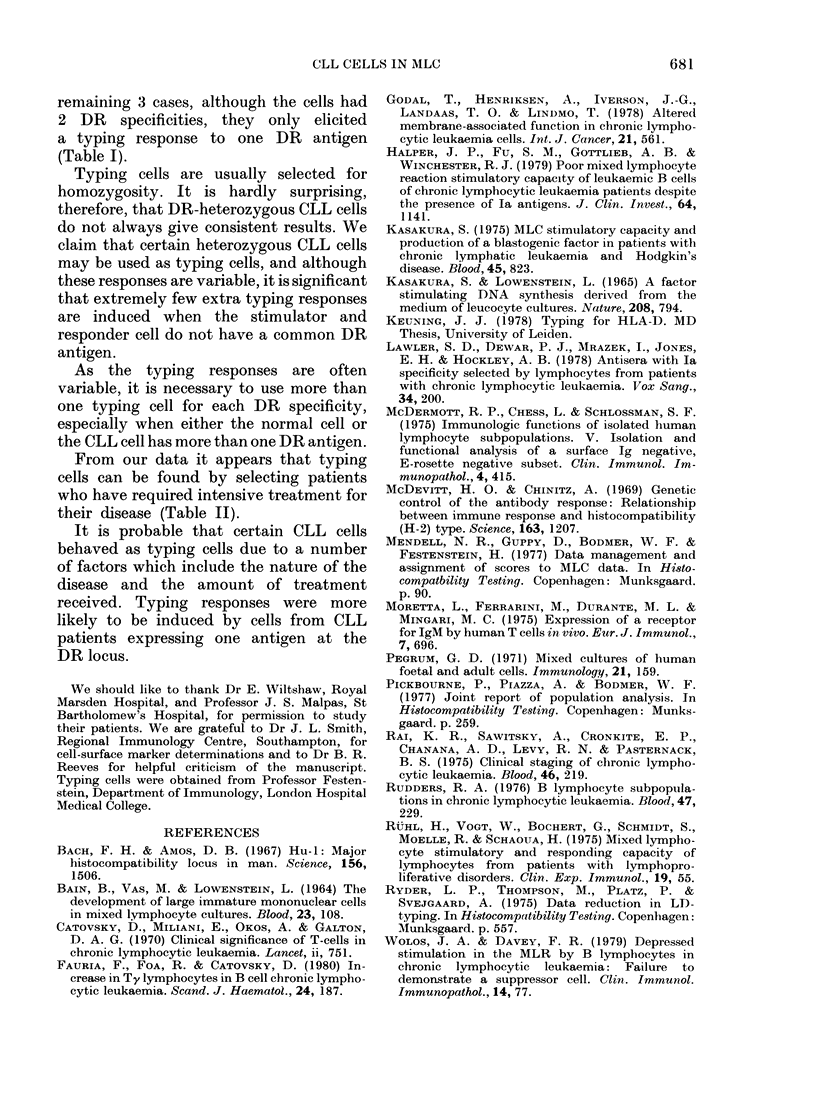# Stimulation by CLL cells in mixed lymphocyte culture (MLC).

**DOI:** 10.1038/bjc.1981.253

**Published:** 1981-11

**Authors:** E. H. Jones, A. B. Hockley, S. D. Lawler

## Abstract

Cells from patients with chronic lymphocytic leukaemia (CLL) do not respond in the mixed lymphocyte culture (MLC) but are able to stimulate the response of normal lymphocytes. Mixed lymphocyte cultures were performed using cells from 24 patients with CLL and cells from 16 normal donors. The stimulatory capacity of 8 of these CLL cells was reduced when a common DR antigen was shared with the normal responding cell. We suggest that cells from certain selected CLL patients may be used in the mixed-lymphocyte reaction for determining the D-locus specificity of normal donors. CLL cells when expressed 1 DR antigen only, induced more clearly defined typing responses than cells with 2 DR antigens. There was no correlation between the ability of a CLL cell to induce a typing response and the T-cell status of the patient. However, a correlation with clinical course was suggested, because most cells which induced a typing response were obtained from patients who had received intensive treatment for the disease.


Br. J. Cancer (1981) 44, 675

### STIMULATION BY CLL CELLS IN MIXED LYMPHOCYTE CULTURE (MLC)

E. H. JONES, A. B. HOCKLEY AND S. D. LAWLER

*From the Department of Cytogenetics and Immunogenetics, Institute of Cancer Research, The Royal Marsden Hospital, London SW3 6JJ*

Received 12 December 1980 Accepted 24 July 1981

**Summary.—Cells from patients with chronic lymphocytic leukaemia (CLL) do not respond in the mixed lymphocyte culture (MLC) but are able to stimulate the response of normal lymphocytes.**

**Mixed lymphocyte cultures were performed using cells from 24 patients with CLL and cells from 16 normal donors. The stimulatory capacity of 8 of these CLL cells was reduced when a common DR antigen was shared with the normal responding cell.**

**We suggest that cells from certain selected CLL patients may be used in the mixed-lymphocyte reaction for determining the D-locus specificity of normal donors. CLL cells which expressed 1 DR antigen only, induced more clearly defined typing responses than cells with 2 DR antigens.**

**There was no correlation between the ability of a CLL cell to induce a typing response and the T-cell status of the patient. However, a correlation with clinical course was suggested, because most cells which induced a typing response were obtained from patients who had received intensive treatment for the disease.**

In MAMMALIAN SPECIES there is a region in the major histocompatibility complex (MHC) concerned with the regulation of immune responses (McDevitt & Chinitz, 1969), called in man, HLA. This region also codes for the Ia antigens which are expressed on B, but not on T lymphocytes (unless activated), and in man they are controlled by the DR locus. Another function of this genetic region, controlled by the D locus, concerns recognition and response in the mixed lymphocyte culture (MLC) (Bach & Amos, 1967). When two populations of normal lymphocytes are mixed together *in vitro* they stimulate and respond to each other and enter DNA synthesis (Bain *et al.*, 1964). In order to study the recognition and response of normal lymphocytes, one population is allowed to remain capable of cell division (the responder population) and the other (the stimulator population) is treated by X-irradiation or mitomycin C so that the cells cannot divide though they remain antigenically intact (Kasakura & Lowenstein, 1965).

In mixed lymphocyte cultures B cells act as stimulator cells and response is the prerogative of T cells (McDermott *et al.*, 1975). It has been found that lymphocytes of patients with chronic lymphocytic leukaemia (CLL) provide an excellent source of cells for B-cell serology. The reactions with anti-DR sera are superior to those given by enriched suspensions of normal B cells (Lawler *et al.*, 1978). We therefore decided to investigate the behaviour of lymphocytes of CLL patients as stimulators in the mixed lymphocyte culture.

CLL cells cannot be used as responders in the mixed lymphocyte culture, so their D-locus specificities cannot be determined, but the correlation with the DR specificities means that the corresponding D type can be deduced in most cases. Our intention was to see whether the stimulatory capacity of these CLL cells in mixed lymphocyte cultures could be correlated with DR specificities and, if so, whether they could be used in typing for D-locus specificities. MLC-defined D-locus specificities correlate with serologically determined DR specificities for the antigens we studied (Pickbourne *et al.*, 1977). In pilot studies, briefly reported at a meeting of the Transplantation Society in November 1978, we found that the stimulating CLL cells showed some specificity. We therefore investigated a number of patients of known clinical status and DR type, to see whether there was any common factor which determined whether the CLL cell could be used for D-locus typing.

### MATERIALS AND METHODS

### *Patients and controls*

Twenty-four patients with chronic lymphocytic leukaemia and 16 normal controls were selected for the experiments on the basis of their DR specificities. These cells had been typed with sera used in the 6th, 7th and 8th Histocompatibility Workshops (1975, 1977, 1980). DW specificities of the normal donors were determined by using homozygous typing cells obtained from recognised typing laboratories through Dr J. L. Sachs of the London Hospital.

All CLL patients were classified at diagnosis and at the time of the MLC, by Rai's staging system (Rai *et al.*, 1975) as follows:

Stage 0—Absolute lymphocytosis ($>15{,}000$ lymphocytes $\times 10^6$/l).

Stage I—Absolute lymphocytosis + enlarged lymph nodes.

Stage II—Absolute lymphocytosis + enlarged liver and/or spleen.

Stage III—Absolute lymphocytosis + anaemia (Hb $<11$ g/dl).

Stage IV — Absolute lymphocytosis + thrombocytopenia (platelet count $<100{,}000 \times 10^6$/l).

### *Techniques*

*Separation of lymphocytes for HLA-A, B, C and DR typing.*—Lymphocytes used for HLA-A, B and C typing were separated from heparinized blood on a Ficoll–Triosil gradient. Suspensions of enriched B lymphocytes for DR typing were separated from heparinized blood by rosetting T lymphocytes with papain-treated sheep erythrocytes following the method of Welsh & Batchelor (1975, personal communication). The suspensions consisted of 70–90% cells assessed by immunofluorescence after staining with fluorescein-labelled anti-human globulin (Wellcome).

*Cytotoxicity assay for HLA-A, B, C and DR antigens.*—Lymphocytes from CLL patients and from normal controls were typed for ABC antigens as follows: 1 $\mu$l of cell suspension ($2\times 10^6$ cells/ml in 50% AB serum: 50% complement-fixation test buffer) was added to 1 $\mu$l antisera in Terasaki plates. The cells were incubated at room temperature for 30 min, 5 $\mu$l rabbit complement was then added and after further incubation at room temperature for 1 h, the percentage of dead cells was estimated by trypan-blue exclusion.

The cytotoxic test used to type CLL cells and B cells from normal controls for DR antigens was similar to the method used for ABC antigens, except that both incubation times were doubled.

*Mixed lymphocyte cultures (MLC).*—Lymphocytes were obtained from fresh heparinized blood samples and separated on a Ficoll–Triosil gradient. Cells from patients with CLL were frozen in 50% autologous plasma and 10% dimethyl sulphoxide, and thawed when required. Normal cells were obtained from donors on the day of culture and were not frozen.

Cultures were performed in triplicate in microtest plates using equal concentrations ($5\times 10^4$) of CLL cells (stimulator cells) and normal lymphocytes (responder cells).

Lymphocytes were suspended in a total volume of 100 $\mu$l of 25mM Hepes-buffered RPMI 1640 (Gibco-Europe Ltd) containing 20% decomplemented pooled AB serum. Culture medium was supplemented with 15mM sodium bicarbonate, 2mM glutamine, penicillin (100,000 iu/l), and streptomycin (100 mg/l). 0·5 $\mu$Ci [$^3$H]dT (5 Ci/mmol) (Radiochemical Centre, Amersham) were added to each culture after 5 days. After a further 16 h the cultures were harvested in a Minimash (Dynatech) and incorporation of [$^3$H]dT was measured by liquid scintillation counting in a Packard autospectrometer.

*Evaluation of MLC results.*—MLC data expressed as ct/min were converted into a "score" by calculation of "double normalized values" based on the method of Ryder *et al.* (1975) and Mendell *et al.* (1977).

A double normalized value of 50 or less was the figure selected by us to indicate a typing response.

*Lymphocyte marker studies.*—Fresh cells from the CLL patients were examined for the presence of the following receptor sites: Fc$\gamma$; C3; SIgG, A, M, D ($\kappa$ and $\lambda$). These investigations were carried out by Dr J. L. Smith, Regional Immunology Centre, Southampton.

The percentage of T$\gamma$ lymphocytes was assessed in 23 CLL patients by the method of Moretta *et al.* (1975). The protocol and antiserum were kindly supplied to us by Professor J. R. Humphrey, Royal Postgraduate Medical School.

### RESULTS

### *Mixed lymphocyte cultures*

We decided that a CLL cell could be regarded as a typing cell if mixed lymphocyte reactivity, expressed as the double normalized value, was 50 or below in most tests. The CLL cells were classified as typing cells if the mixed lymphocyte reactivity was reduced when there was a common DR antigen between the CLL cell and the normal cell. The CLL cells were regarded as non-typing cells if there was no reduction in mixed lymphocyte

TABLE I.—*MLC response between CLL cells (stimulator) and normal cells (responder) expressed as median ct/min and double normalized values (DNV)*

| Normal control (responder) | | | CLL (stimulator) | | | | | | | |
|---|---|---|---|---|---|---|---|---|---|---|
| | | | GW | AC | MW | TK | JP | MD | EP | JM |
| | | DR | 2 | 2,3 | 2,3 | 2,3 | 3 | 4 | 7 | 7 |
| | DR | Background ct/min | (379) | (311) | (629) | (201) | (240) | (330) | (449) | (648) |
| NB | 1 | (1,500) | 24,514† | 51,011 | 17,286 | 14,257 | 23,842 | 60,596 | 42,392 | 32,864 |
| | | | 100* | 102·3 | 114 | 104·6 | 58·9 | 53·9 | 100 | 83·1 |
| JQ | 2 | (628) | 708 | 31,396 | 173 | ND | 27,919 | ND | 28,370 | ND |
| | | | **21·4** | **41·1** | **13·9** | | 103·2 | | 100 | |
| EJ | 2 | (2,009) | 4,581 | 15,748 | 4,983 | ND | 31,500 | 101,684 | 40,112 | ND |
| | | | **21·6** | **39·1** | **42·2** | | 89·6 | 104·3 | 108·9 | |
| GE | 2,1 | (2,041) | 5,361 | 6,853 | 751 | ND | 17,368 | 43,030 | 10,081 | ND |
| | | | 58·5 | **39·5** | **14·6** | | 114·5 | 100 | 63·5 | |
| VS | 2,3 | (2,183) | 1,699 | 5,120 | 1,272 | 1,132 | 3,821 | 32,812 | 8,495 | 14,638 |
| | | | **22** | **41·8** | **35·4** | **16·4** | **35·7** | 110·6 | 75·9 | 73·6 |
| JS | 2,3 | (1,783) | 1,551 | 4,445 | 1,206 | 2,347 | ND | ND | 10,568 | 7,402 |
| | | | 61·2 | 53·4 | **47·6** | 103·3 | | | 117·3 | 112·4 |
| SL | 2,4 | (1,812) | 2,148 | 15,000 | 2,721 | 1,368 | 16,040 | 17,612 | 24,093 | 21,878 |
| | | | **16·9** | 52 | **31·6** | **17·2** | 76·3 | **30·2** | 109·5 | 95·9 |
| RF | 3 | (610) | 13,226 | 37,454 | 4,432 | 2,075 | ND | ND | 23,098 | 31,654 |
| | | | 113·3 | 97·5 | **38·4** | **19·5** | | | 55·4 | 104·0 |
| GD | 3 | (3,032) | 19,048 | 21,805 | 6,591 | ND | 8,445 | 31,625 | 20,054 | ND |
| | | | 136·7 | 82·6 | 85 | | **36·7** | **49·5** | 83·2 | |
| AH | 3 | (3,832) | 14,401 | 26,250 | 12,504 | 8,032 | ND | ND | 52,539 | 32,219 |
| | | | 99 | 54·8 | 85·9 | 61·3 | | | 101·2 | 84·9 |
| LM | 3,7 | (1,531) | 14,633 | 30,543 | 13,678 | ND | 8,528 | 49,216 | 29,808 | ND |
| | | | 56·6 | 62·5 | 95·2 | | **19·9** | **41·6** | 66·8 | |
| EN | 3,7 | (894) | 2,210 | 14,975 | 1,410 | ND | 3,484 | 26,314 | 10,042 | ND |
| | | | **28** | 100 | **32** | | **26·7** | 72·6 | 73·5 | |
| LN | 4 | (2,878) | 23,475 | 45,487 | 19,263 | ND | 41,606 | 41,942 | 35,700 | ND |
| | | | 106·1 | 108·5 | 156·1 | | 113·6 | **41·3** | 93·2 | |
| JT | 4,5 | (1,819) | 3,763 | 40,370 | 10,252 | 12,346 | ND | ND | 32,438 | 28,271 |
| | | | **26·5** | 86·5 | 72·3 | 96·8 | | | 64·1 | 70·2 |
| DK | 7 | (3,716) | 11,285 | 33,372 | 9,821 | 3,091 | 29,134 | 69,058 | 9,199 | 12,954 |
| | | | 101·1 | 100 | 100 | **30·8** | 100 | 85·5 | **30·2** | **44·5** |
| KG | 7,2 | (600) | 3,069 | 26,206 | 3,052 | 1,811 | ND | ND | 12,043 | 10,866 |
| | | | **33** | 86·1 | **32·8** | **21·6** | | | **36·5** | **45** |

† Median ct/min.
* Double normalized value. DNV of 50 or less in bold type.
ND = MLC not performed.

TABLE II.—*HLA type, clinical and haematological data of CLL patients*

| Name | Typing response for DW in MLC | HLA A Locus | HLA B Locus | HLA C Locus | HLA DR Locus | Sex | Age at diagnosis (years) | Dead/ alive | Survival (mths) | Treatment† | At time of MLC: White cell count ($\times 10^9$/l) | At time of MLC: Lymphocytes (%) | At time of MLC: T lymphocytes (%) | At time of MLC: Tγ lymphocytes (%) | At time of MLC: Cell surface immunoglobulin markers | Staging at diagnosis (Rai) | Staging at time of MLC (Rai) |
|---|---|---|---|---|---|---|---|---|---|---|---|---|---|---|---|---|---|
| Typing cells (8) | | | | | | | | | | | | | | | | | |
| GW | 2 | 3,11 | 7,40 | — | 2 | F | 49 | A | 70 | None | 32 | 89 | 14 | 21 | M.Dk | 0 | I |
| AC | 2 | 1,3 | 7,8 | — | 2,3 | M | 71 | D | 72 | P.CL. | 12 | 94 | 16 | 17 | M.Dk | IV | NS |
| MW | 2 | 1,3 | 7,8 | — | 2,3 | F | 83 | D | 72 | CY.P.CL. | 148 | 90 | 7 | 9 | (Mk) | IV | IV |
| TK | 2 | 1,28 | 8,40 | 3 | 2,3 | M | 65 | D | 64 | P.CL.CY.VCR.P.L. | 6 | 28 | 4 | ND | ND | II | NS |
| JP | 3 | 1 | 8 | — | 3 | M | 58 | D | 94 | CY.P.CL.M.VCR.PR. | 11 | 90 | 5 | 21 | D.Mk | II | II |
| MD | 4 | 2 | W44 | 5 | 4 | M | 45 | A | 62 | P.CL.VCR.A.CY. | 27 | 78 | 17 | 35 | (Mk)§ | II | II |
| EP | 7 | 2,3 | 40,W44 | — | 7 | F | 74 | D | 144 | X.P. | 31 | 80 | 14 | 28 | Dk | I | 0 |
| JM | 7 | 2,3 | 15,W35 | 3,4 | 7 | M | 63 | D | 36 | CL.VCR.BL. | 23 | 92 | 14 | 9 | M.Dλ | NS | I |
| | | | | | | | | | | Mean | 36·2 | 80·1 | 11·3 | 20·0 | | | |
| | | | | | | | | | | Range | 6–148 | 28–94 | 4–17 | 9–35 | | 0–IV | 0–IV |
| Non-typing cells (16) | | | | | | | | 4 D | | 5 untreated | | | | | | | |
| | | | | | | | | 12 A | | 11 treated | | | | | | | |
| | | | | | | | | | | Mean | 41·9 | 73·2 | 6·8 | 25·8 | | | |
| | | | | | | | | | | Range | 7–206 | 26–100 | 1–16 | 9–39 | | 0–IV | 0–IV |

ND = Not done.
NS = Not stageable (lymphocyte count less than $15{,}000 \times 10^6$/l).
§ () = weak immunoglobulin.
† Treatment: A, allopurinol; BL, bleomycin; CL, chlorambucil; CY, cyclophosphamide; L, Leo 1031 (chlorambucil + steroid); M, mustine; P, prednisolone; PR, procarbazine; VCR, vincristine; X, irradiation.

reactivity when the responder cell shared a DR antigen.

The 24 CLL cells tested were thus classified into two categories; 16 of these did not induce typing responses, but as shown in Table I, cells from 8 of the patients did induce a consistent typing response.

Table I shows the response in the MLC between responder (normal cell) and the stimulator cell (CLL), expressed as median ct/min and double normalized values (DNV). The DNV of 50 or less are in bold type, indicating a significant typing response. Five of the CLL cells expressed one DR antigen only (GW, JP, MD, EP, JM). Three of the CLL cells expressed two DR antigens (AC, MW, TK).

*Typing response for DR2*

The CLL cell GW typed for DR2, and stimulated normal cells lacking DR2. Both the normal cells expressing the DR2 antigen only (JQ, EJ) and 3/5 cells heterozygous for DR2 (VS, SL, KG) showed a typing response.

*DR3*.—Similarly the CLL cell JP typed for DR3, and stimulated normal cells lacking DR3. Both the normal cells expressing the DR3 antigen only (GD) and 3/4 cells heterozygous for DR3 (VS, LM, EN) showed a typing response.

*DR4*.—The CLL cell MD typed for DR4, and stimulated normal cells lacking DR4. The normal cell expressing DR4 only (LN) and one heterozygous for DR4 (SL), showed a typing response.

*DR7*.—The CLL cells EP and JM typed for DR7 and stimulated normal cells lacking DR7. The normal cells expressing DR7 only (DK and KG) showed a typing response.

Where the stimulator and responder cell did not share a common DR antigen, the CLL cell rarely induced a typing response. The CLL cell GW typing for DR2 showed two extra reactions with $DR2^-$ normal cells, and CLL cell MD typing for DR4 showed two extra typing responses with $DR4^-$ cells. The cells JE, EP and JM did not induce any extra typing responses.

The 3 CLL cells which expressed both DR2 and DR3 antigens gave a specific typing response for DR2 only, and gave poor typing responses for DR3. The normal cells expressing DR2 only (JQ, EJ), and 2/5 cells heterozygous for DR2 (GE, VS), showed a typing response to the CLL cell AC (DR2, 3). The normal cells expressing DR2 only (JQ, EJ) and all 5 cells heterozygous for DR2 (GE, VS, JS, SL, KG) showed a typing response to the CLL cell MW (DR2, 3). Three of the 4 normal cells heterozygous for DR2 (VS, SL, KG) showed a typing response to the CLL cell TK (DR2, 3). The CLL cells AC and MW did not induce extra typing responses and the CLL cell TK induced one extra typing response to a $DR2^-$ responder cell.

Table II shows the HLA types and summarizes the clinical and haematological data of the 8 CLL patients whose cells could be used as D-locus typing cells. The results summarize the success of the CLL cell as an inducer of typing responses, in relation to the DR type of the stimulator and responder cell. Out of a total of 8 typing cells, 2 (MD, JP) expressed 1 HLA-A and 1 HLA-B locus antigen and 5 (MD, JP, GW, EP and JM) expressed 1 HLA-DR antigen only. CLL cells with one antigen at the DR locus induced more clearly defined typing responses than those with 2 DR antigens. It appears that the expression of 1 A and 1 B locus antigen is not a prerequisite for a typing response.

Comparing the 8 CLL patients whose cells behaved as typing cells with the group of 16 patients whose cells did not, there was no difference in the mean white cell count, percentage of T lymphocytes or percentage of T$\gamma$ lymphocytes. Staging of the patients at diagnosis and at the time of the MLC revealed little difference in the Rai classification in the two groups.

### DISCUSSION

The behaviour of CLL cells as stimulators and responders in MLC has been studied previously. Cells from patients with CLL have been used as stimulators in mixed cell cultures with foetal liver and thymus (Pegrum, 1971) and with allogeneic normal lymphocytes (Rühl *et al.*, 1975). The response of B lymphocytes from CLL patients to B cell mitogens is impaired (Godal *et al.*, 1978). B cells from CLL patients are thought to have either an unimpaired stimulatory capacity in the mixed lymphocyte reaction (Kasakura, 1975) or a decreased stimulatory capacity (Wolos & Davey, 1979; Halper *et al.*, 1979). However, in our experiments, calculation of the stimulator index for all CLL cells used indicated that these were effective stimulators provided they did not share a common DR antigen with the normal responder cell. It has been suggested that optimal stimulation in the MLC may be obtained by increasing the concentration of CLL cells to $1{\cdot}25$–$2{\cdot}5\times10^6$/ml (Halper *et al.*, 1979). In our experiments we found that maximum stimulation of normal responder cells occurred at a concentration of $0{\cdot}5\times10^6$/ml stimulator cells, the mixed-lymphocyte reaction being reduced at a lower concentration ($0{\cdot}25\times10^6$/ml) or a higher concentration ($10^6$/ml).

Other workers, though commenting on the poor response induced by CLL cells as stimulators, did not relate their findings to the D or DR specificities of the responding cells (Kasakura, 1975).

Although not all the 16 CLL cells in our study showed specificity in their stimulating capacity, 8 of them elicited a specific typing response. This was sufficiently reliable for us to suggest the possibility of using these cells for determining the D-locus specificity of normal donors. There was no difference in median ct/min or DNV between the group of cells which induced a typing response and the group of non-typing cells. This indicates that the typing effect was produced by a factor other than an overall reduction in median ct/min or a reduction in stimulatory capacity of the CLL cell.

We carefully examined the clinical and haematological data of CLL patients, to see whether any factors determined which CLL cells could be used as a typing cell.

It has been suggested by Catovsky *et al.* (1970) that although the percentage of T lymphocytes is similar in treated and untreated CLL patients, the increased absolute number of T cells may have a clinical significance.

We considered whether the percentage of T cells in individual patients could be correlated with the ability to give a typing response. The percentage of T cells in individual patients varied between 17% and 40%, and there was no evidence that the ability to induce a typing response could be correlated with either the percentage of T cells or the mean T-cell count (Table II).

In CLL an increase in the proportion and absolute number of $T\gamma$ suppressor lymphocytes has been observed (Fauria *et al.*, 1980). We considered whether an increased number of T suppressor cells could account for the ability of certain CLL cells to give a typing response, but as there was no difference in the $T\gamma$ cells between the two groups, this was thought unlikely.

Two sub-populations of B cells have been defined in CLL (Rudders, 1976). It appears unlikely that the ability of CLL cells to induce a typing response is related to the sub-population class, as all except one of the cases (FF) in this study were classified as typical cells with polyclonal immunoglobulin and a lack of intracellular immunoglobulin.

It appears that certain carefully selected cells from patients with CLL can be used to type for D locus specificities. The scarcity of homozygous cells is a major disadvantage of D-locus typing. Keuning (1978) observed that only 22 offspring from 209 consanguineous marriages were homozygous at the HLA-D locus. Therefore, substitution of CLL cells for D-locus typing could be very useful.

To summarize our results: among 8 CLL cells that showed a typing response, 2 expressed one antigen only at the HLA-A, B and DR loci, and another 3 had only one DR specificity. In the remaining 3 cases, although the cells had 2 DR specificities, they only elicited a typing response to one DR antigen (Table I).

Typing cells are usually selected for homozygosity. It is hardly surprising, therefore, that DR-heterozygous CLL cells do not always give consistent results. We claim that certain heterozygous CLL cells may be used as typing cells, and although these responses are variable, it is significant that extremely few extra typing responses are induced when the stimulator and responder cell do not have a common DR antigen.

As the typing responses are often variable, it is necessary to use more than one typing cell for each DR specificity, especially when either the normal cell or the CLL cell has more than one DR antigen.

From our data it appears that typing cells can be found by selecting patients who have required intensive treatment for their disease (Table II).

It is probable that certain CLL cells behaved as typing cells due to a number of factors which include the nature of the disease and the amount of treatment received. Typing responses were more likely to be induced by cells from CLL patients expressing one antigen at the DR locus.

We should like to thank Dr E. Wiltshaw, Royal Marsden Hospital, and Professor J. S. Malpas, St Bartholomew's Hospital, for permission to study their patients. We are grateful to Dr J. L. Smith, Regional Immunology Centre, Southampton, for cell-surface marker determinations and to Dr B. R. Reeves for helpful criticism of the manuscript. Typing cells were obtained from Professor Festenstein, Department of Immunology, London Hospital Medical College.

### REFERENCES

BACH, F. H. & AMOS, D. B. (1967) Hu-1: Major histocompatibility locus in man. *Science*, **156**, 1506.

BAIN, B., VAS, M. & LOWENSTEIN, L. (1964) The development of large immature mononuclear cells in mixed lymphocyte cultures. *Blood*, **23**, 108.

CATOVSKY, D., MILIANI, E., OKOS, A. & GALTON, D. A. G. (1970) Clinical significance of T-cells in chronic lymphocytic leukaemia. *Lancet*, ii, 751.

FAURIA, F., FOA, R. & CATOVSKY, D. (1980) Increase in Tγ lymphocytes in B cell chronic lymphocytic leukaemia. *Scand. J. Haematol.*, **24**, 187.

GODAL, T., HENRIKSEN, A., IVERSON, J.-G., LANDAAS, T. O. & LINDMO, T. (1978) Altered membrane-associated function in chronic lymphocytic leukaemia cells. *Int. J. Cancer*, **21**, 561.

HALPER, J. P., FU, S. M., GOTTLIEB, A. B. & WINCHESTER, R. J. (1979) Poor mixed lymphocyte reaction stimulatory capacity of leukaemic B cells of chronic lymphocytic leukaemia patients despite the presence of Ia antigens. *J. Clin. Invest.*, **64**, 1141.

KASAKURA, S. (1975) MLC stimulatory capacity and production of a blastogenic factor in patients with chronic lymphatic leukaemia and Hodgkin's disease. *Blood*, **45**, 823.

KASAKURA, S. & LOWENSTEIN, L. (1965) A factor stimulating DNA synthesis derived from the medium of leucocyte cultures. *Nature*, **208**, 794.

KEUNING, J. J. (1978) Typing for HLA-D. MD Thesis, University of Leiden.

LAWLER, S. D., DEWAR, P. J., MRAZEK, I., JONES, E. H. & HOCKLEY, A. B. (1978) Antisera with Ia specificity selected by lymphocytes from patients with chronic lymphocytic leukaemia. *Vox Sang.*, **34**, 200.

MCDERMOTT, R. P., CHESS, L. & SCHLOSSMAN, S. F. (1975) Immunologic functions of isolated human lymphocyte subpopulations. V. Isolation and functional analysis of a surface Ig negative, E-rosette negative subset. *Clin. Immunol. Immunopathol.*, **4**, 415.

MCDEVITT, H. O. & CHINITZ, A. (1969) Genetic control of the antibody response: Relationship between immune response and histocompatibility (H-2) type. *Science*, **163**, 1207.

MENDELL, N. R., GUPPY, D., BODMER, W. F. & FESTENSTEIN, H. (1977) Data management and assignment of scores to MLC data. In *Histocompatbility Testing*. Copenhagen: Munksgaard. p. 90.

MORETTA, L., FERRARINI, M., DURANTE, M. L. & MINGARI, M. C. (1975) Expression of a receptor for IgM by human T cells *in vivo*. *Eur. J. Immunol.*, **7**, 696.

PEGRUM, G. D. (1971) Mixed cultures of human foetal and adult cells. *Immunology*, **21**, 159.

PICKBOURNE, P., PIAZZA, A. & BODMER, W. F. (1977) Joint report of population analysis. In *Histocompatibility Testing*. Copenhagen: Munksgaard. p. 259.

RAI, K. R., SAWITSKY, A., CRONKITE, E. P., CHANANA, A. D., LEVY, R. N. & PASTERNACK, B. S. (1975) Clinical staging of chronic lymphocytic leukaemia. *Blood*, **46**, 219.

RUDDERS, R. A. (1976) B lymphocyte subpopulations in chronic lymphocytic leukaemia. *Blood*, **47**, 229.

RÜHL, H., VOGT, W., BOCHERT, G., SCHMIDT, S., MOELLE, R. & SCHAOUA, H. (1975) Mixed lymphocyte stimulatory and responding capacity of lymphocytes from patients with lymphoproliferative disorders. *Clin. Exp. Immunol.*, **19**, 55.

RYDER, L. P., THOMPSON, M., PLATZ, P. & SVEJGAARD, A. (1975) Data reduction in LD-typing. In *Histocompatibility Testing*. Copenhagen: Munksgaard. p. 557.

WOLOS, J. A. & DAVEY, F. R. (1979) Depressed stimulation in the MLR by B lymphocytes in chronic lymphocytic leukaemia: Failure to demonstrate a suppressor cell. *Clin. Immunol. Immunopathol.*, **14**, 77.